# The manipulator behind “Scissors”: γ -secretase and its modulators in Alzheimer’s disease

**DOI:** 10.3389/fnagi.2025.1637671

**Published:** 2025-08-25

**Authors:** Gao Ning, Xing Fan, Du Juan, Zhao wenxue, Wang Sijia, Chen Meinei, Dong Xiaolong, Qi Yiming

**Affiliations:** ^1^Yan’an Medical College of Yan’an University, Yan’an, Shaanxi, China; ^2^Yan’an Key Laboratory of Northern Shaanxi Tumor Prevention and Treatment, Yan’an Medical College, Yan’an University, Yan’an, Shaanxi, China

**Keywords:** γ-secretase, Alzheimer’s disease, GSMS, GSMPs, type I transmembrane proteins

## Abstract

The intramembrane aspartic protease, γ-secretase, is a heterotetrameric protein complex composed of four integral membrane proteins: presenilin (PSEN), nicastrin (NCT), Anterior pharynx defective-1 (APH-1), and presenilin enhancer 2 (PEN-2). These components are sequentially assembled into a functional complex. γ-secretase is ubiquitously expressed in all cells and tissues and exhibits enzymatic activity akin to “molecular scissors” by cleaving various type I transmembrane proteins. The primary substrates of this complex include amyloid precursor protein (APP) and Notch. The role of APP in the pathogenesis of Alzheimer’s disease (AD) has been extensively investigated. Although γ-secretase inhibitors (GSIs) have been evaluated for their therapeutic potential in AD, their clinical application is limited due to significant toxic side effects. Recently, γ-secretase modulators (GSMs) have emerged as promising alternatives, offering new opportunities for the treatment of AD, especially the inherent γ-secretase modulatory proteins (GSMPs) within cells. Research on GSMPs has ushered in a new era for mitigating the side effects of AD drugs. In this review, we systematically summarize recent advancements in the study of γ-secretase in relation to AD and provide an overview of GSMs and GSMPs, thereby offering potential insights for the development of therapeutic strategies for AD.

## 1 Introduction

**FIGURE 1:**
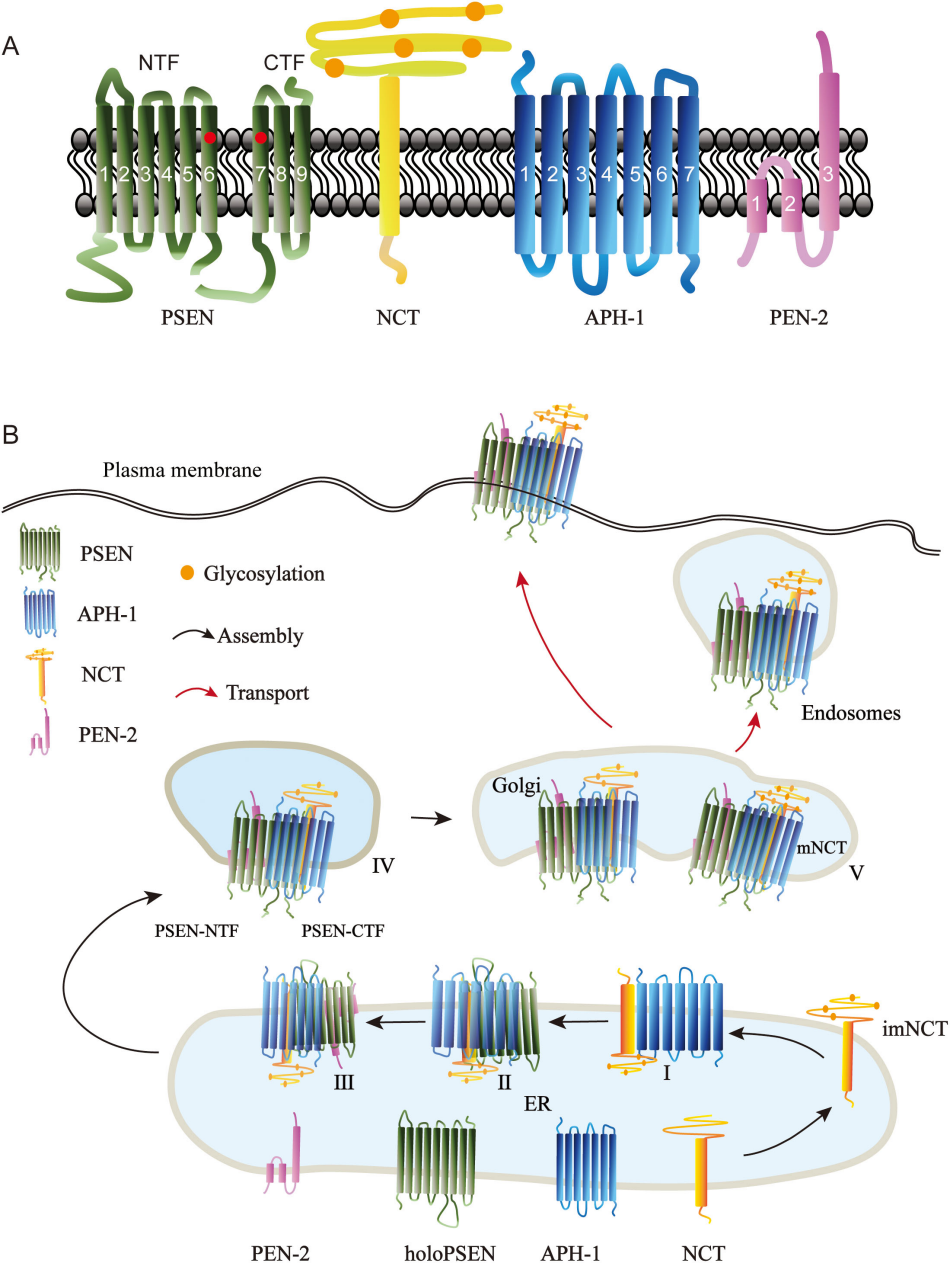
Structure of γ-secretase subunits and their assembly in cells. **(A)** The γ-secretase complex is composed of four multipass membrane proteins, presenilin (PSEN), nicastrin (NCT), Anterior pharynx defective-1 (APH-1), and presenilin enhancer 2 (PEN-2). PSEN represents the catalytic components in the complex; it consists of nine transmembrane helices, including two catalytic aspartate residues (circled in red) in TM6 and TM7. PSEN is cleaved endoproteolytically between TM6 and TM7 to produce amino terminal fragment (NTF) and carboxyl terminal fragment (CTF). NCT is a cofactor of PSEN; it is a type I transmembrane glycoprotein with a large glycosylated extracellular domain (orange circles indicate the glycosalation site). APH-1 is a seven-transmembrane protein required for APH-1/NCT trafficking to the cell surface (Goutte et al., 2002). PEN-2, the smallest component of the -secretase complex, modulates the activity of -secretase. PEN-2 has three membrane-embedded domains, the first two domains traversing only half of the lipid bilayer. **(B)** The four γ-secretase subunits are biosynthesized in the endoplasmic reticulum (ER) and taking a stepwise manner to form a complex. Subcomplex I: N-glycosylated Nicastrin forms imNCT, which then binds to APH-1 to create imNCT/APH-1. PSEN holoprotein binds to the imNCT/APH-1 subcomplex. Subcomplex III: PEN-2 is fused to imNCT/APH-1/PSEN holoprotein. Subcomplex IV: PSEN autoproteolysis at theTMD6 and TMD7 producing PSEN-NTF and PSEN-CTF fragments. Subcomplex V: Subcomplex IV is transported to the Golgi, where NCT undergoes further N-glycosylation to generate mature Nicastrin. Then, the mature γ-secretase complex moves on toward the plasma membrane and endosomes.

The γ-secretase complex is an intramembrane-cleaving protease (I-CliP) that catalyzes the intramembrane domain (TMD) of its protein substrates and performs a wide variety of essential biological roles (Wolfe, 2019). The γ-secretase has been identified as a heterotetrameric complex consisting of four membrane protein subunits: APH-1, NCT, PSEN, and PEN-2 (Oikawa and Walter, 2019; Figure 1A). It takes a stepwise manner to assemble the four subunits into one complex, the correct assembly of the γ-secretase is tightly regulated, and then the mature γ-secretase complex is transported to the plasma membrane and endosomes to perform its functions (Dries and Yu, 2008; Smolarkiewicz et al., 2013; Figure 1B). Alzheimer disease (AD) is a common cause of cognitive impairment in middle-aged and elderly people. It is a hereditary and sporadic neurodegenerative disorder (Knopman et al., 2021). Excessive phosphorylation of tau protein (Anwar et al., 2024; Adnan et al., 2023), and deposition of beta-amyloid protein (Aβ) are currently the mainstream theories recognized as causing AD (Wolfe, 2024). γ-secretase has various substrates, in AD, γ-secretase produces Aβ by cleaving amyloid precursor protein (APP). The abnormal accumulation and aggregation of these peptides are considered crucial factors in the pathological changes of AD (Kim et al., 2024). Despite the key role γ-secretase plays in AD pathogenesis (Hakem et al., 2024), direct inhibition strategies have not achieved satisfactory outcomes in clinical trials, largely because inhibiting multiple γ-secretase substrates may disrupt normal physiological functions (Dries and Yu, 2021; Caldwell et al., 2022). As a result, researchers are investigating alternative approaches to regulate γ-secretase activity, such as the creation of GSMs, which represent a class of small molecules capable of influencing the activity of γ-secretase (Wolfe, 2024; Hakem et al., 2024). Unlike GSIs, which directly suppress the enzyme’s activity, GSMs modify the cleavage patterns of substrates via allosteric regulation. This process leads to a reduction in the generation of harmful amyloid peptides (Rynearson et al., 2021; Petit et al., 2022). Furthermore, the design of GSMs aims to minimize side effects associated with GSIs observed during clinical trials, such as interference with the Notch signaling pathway (Hur, 2022). In recent years, the growing comprehension of γ-secretase’s structure and function has enhanced our knowledge of GSM mechanisms. Cryo-electron microscopy has played a pivotal role in mapping the structural configuration of γ-secretase when it interacts with GSMs, revealing critical details about how GSMs influence the γ-secretase complex (Dries and Yu, 2021; Yang et al., 2021). Studies on GSMs can help to improve our targeted therapy for AD.

The activity of γ-secretase is modulated by a variety of factors, including the properties of its substrates and the intracellular microenvironment. In addition to APP, many γ-secretase substrates are associated with AD, such as Notch, CD43, CD44, CD91, CD269, CSF-1R, etc., The study of these substrates related to AD may give us new ideas about whether they regulate the activity of γ-secretase to act together in AD. There is evidence that the enzymatic activity of γ-secretase can also be influenced by interferon-induced transmembrane protein 3 (IFITM3), which is upregulated in tissue samples from certain late-onset Alzheimer’s disease patients and exhibits a positive correlation with γ-secretase activity (Hur et al., 2020). Several studies have demonstrated that inhibiting γ-secretase-activating protein (GSAP) effectively reduces Aβ production while leaving other critical γ-secretase substrates unaffected (Kim et al., 2024). Apolipoprotein E (ApoE), the most prominent genetic risk factor for sporadic Alzheimer’s disease (sAD), interacts with γ-secretase and selectively inhibits its activity in a substrate-dependent manner (Hou et al., 2023). These intracellular proteins, which regulate γ-secretase, are referred to as γ-secretase modulatory proteins (GSMPs). If GSMPs can be effectively modulated to achieve therapeutic effects for AD, this approach may potentially further mitigate the side effects associated with chemical drugs. In summary, γ-secretase and its substrates are intricately associated with the onset and progression of AD. A deeper investigation into the underlying mechanisms is crucial for advancing AD treatment. Notably, targeted therapies involving GSMs and GSMPs hold promise in providing new hope for AD patients.

## 2 Subunits of γ-secretase and their assembly

### 2.1 Subunits of γ-secretase

#### 2.1.1 PSEN

Early in the 1990’s, two human early-onset familial Alzheimer’s disease (EOFAD) -related loci were discovered on chromosomes 14 (Rogaev et al., 1995) and 1 (Schellenberg et al., 1992). These loci were subsequently identified as two homologous genes: *Presenilin1* (*psen1*) and *Presenilin2* (*psen2*) (Levy-Lahad et al., 1995; Sherrington et al., 1995). PSEN are the catalytic components with aspartyl protease activity, and more than 150 AD causing mutations have been reported in both genes, with the majority occurring in PSEN1 (Zhang et al., 2014). The full-length PSEN consists of nine transmembrane helices (TM1-9) and two catalytic aspartate residues in TM6 (Asp257) and TM7 (Asp385) (Spasic et al., 2006). During γ-secretase complex maturation, the presenilin holoprotein becomes inactive and is rapidly degraded by proteolytic activity. PSEN undergoes autoproteolysis into stable N-terminal fragments (NTF, TM1-6) and C-terminal fragments (CTF, TM7-9) (Fraser et al., 1998; Kim et al., 1997; Thinakaran et al., 1996; Figure 1A). The endoproteolytic fragments of PSEN proteins are predominantly localized in the endoplasmic reticulum (ER) and Golgi apparatus (Annaert et al., 1999), but also found in the plasma membrane (Marambaud et al., 2002), endosomes (Vetrivel et al., 2004), phagosomes (Jutras et al., 2005), lysosomes (Sannerud et al., 2016), mitochondria (Area-Gomez and Schon, 2016; Area-Gomez et al., 2009), and nuclear envelope (Kimura et al., 2001). where they are interacted with NCT, APH-1, and PEN-2 to form a 250kDa complex (Yu et al., 1998).

#### 2.1.2 NCT

A novel protein (Nicastrin, NCT) was found in 2000 by immunoprecipitation of PSEN1 combined with mass spectrometry (Yu et al., 2000). *Nicastrin* was named after the Italian village of Nicastro, where the PSEN-associated forms of Familial Alzheimer’s disease were discovered (Feldman et al., 1963; Foncin et al., 1985). Human *NCT* gene maps to chromosomee 1, it has been identified as risk genes for Alzheimer’s disease susceptibility locus in two genome surveys (Zubenko et al., 1998; Owen et al., 2000). NCT is a type I transmembrane glycoprotein with 709 amino acids, a putative amino-terminal signal peptide, an N-terminal hydrophilic domain, N-myristoylation and phosphorylation motifs, a transmembrane domain, and a 20-residue hydrophilic carboxy terminus (Yu et al., 2000; Figure 1A). The conserved functions of NCT for Notch signaling have been revealed from studies in *Caenorhabditis. Elegans* (Goutte et al., 2000), *Drosophila melanogaster* (Chung and Struhl, 2002; Hu et al., 2002), and mice (Beher et al., 2003). NCT is the first identified PSEN cofactor, and it plays a critical role in PSEN-mediated processing of APP and notch/glp-1 signaling (Yu et al., 2000). The ectodomain of NCT is proposed to recruit substrates into the γ-secretase complex (Shah et al., 2005; Dries et al., 2009).

#### 2.1.3 APH-1

In the genetic screening of *Notch*, *psen*, and *NCT* in *C. elegans*, two multipass transmembrane proteins, Anterior pharynx defective-1 (APH-1) and Presenilin enhancer-2 (PEN-2), interact strongly with PSEN (SEL-12 in *C. elegans*) and NCT (APH-2 in *C. elegans*); *APH-1* deficiency caused a comparable phenotype to *psen* and *NCT* (Francis et al., 2002; Goutte et al., 2002). In mammals, APH-1 is a seven transmembrane protein of approximately 25 kDa (Figure 1A). The GxxxG motif in the fourth TMD of APH-1 is critical for the formation and activation of the γ-secretase complex. APH-1 physically interacts with immature NCT (imNCT) in ER to form a stable subcomplex (LaVoie et al., 2003; Lee et al., 2004), then binds to the C-terminus of PSEN holoprotein (Morais et al., 2003; Steiner et al., 2008). The ternary complex of imNCT/APH-1/holo-PSEN is then transported to the trans-Golgi, where NCT can fully develop and become extensively N-glycosylated. Human APH-1 is encoded by *aph1a* and *aph1b*, of which *aph1a* appears to be the more studied.

#### 2.1.4 PEN-2

The *PEN-2* was identified side-by-side with *APH-1* in a *C. elegans* screening (Francis et al., 2002; Goutte et al., 2002). Human PEN-2 is encoded by a single gene on chromosome 19 (Panmontha et al., 2015). It is the smallest component of γ-secretase with 101 residues, two hydrophobic domains that function as transmembrane spanning domains, and a “U-shaped” structure with the N- and C- termini toward the lumen, according to earlier reports (Crystal et al., 2003; Bergman et al., 2004; Figure 1A). Recent structure research of PEN-2, however, has revealed that there are three membrane-embedded domains, with the C-terminus being luminal and the N-terminus being exposed to the cytoplasm, indicating that the first two domains traverse only half of the lipid bilayer (Zhang et al., 2015). PEN-2 is the last protein to be incorporated into the γ-secretase complex and regulates PSEN auto-hydrolysis and NCT maturation (Francis et al., 2002; Watanabe et al., 2006), and it serves as an ER retention receptor for the immature γ-secretase complex (Fassler et al., 2005). The extracellular region of PEN-2 can interact with the ectodomain of NCT (Sun et al., 2015). The N-terminal extension of PEN-2 can modify the hydrophilic environment of the PSEN catalytic pore (Isoo et al., 2007). Thus, PEN-2 may modulate γ-secretase activity.

### 2.2 Assembly and activation of γ-secretase complex

The correct assembly of the γ-secretase is tightly regulated. It is now widely acknowledged that it takes a stepwise manner to assemble the four subunits into one complex. First, the Nicastrin is synthesized and modified with N-glycosylated in the ER to form imNCT, which then binds to APH-1 to form the heterodimer imNCT/APH-1 subcomplex I (LaVoie et al., 2003). In order to build subcomplex II, the Proximal C-terminus of the PSEN holoprotein connects to the intermediate heterodimer via the TM domain of NCT (Kaether et al., 2005). Then, PEN-2 and TM4 of PSEN are combined to create subcomplex III (Kornilova et al., 2006; Watanabe et al., 2006). However, it is unclear whether this complex is generated by successive binding of PSEN1 and PEN-2 or by a prefabricated PSEN1/PEN-2 subcomplex (Fraering et al., 2004). Subcomplex III is immediately followed by PSEN autoproteolysis at TMD6 and TMD7 to produce PSEN-NTF and PSEN-CTF fragments, which comprise the active form of PSEN inside subcomplex IV (Fukumori et al., 2010). If PEN-2 is not present, the subcomplex is degraded via proteasomes (Prokop et al., 2004). In a next step, approximately 5% of subcomplex IV traffics to the Golgi, where NCT is further N-glycosylated to form mature Nicastrin (mNCT), and this is the subcomplex V (Moniruzzaman et al., 2018), represents the formation of active γ-secretase in the cell. Finally, the mature active γ-secretase complex is then transported to the plasma membrane and endosomes (Dries and Yu, 2008; Smolarkiewicz et al., 2013; Figure 1B). The four-component intramembrane proteinase gamma-secretase is intricately linked to the development of various diseases. Single-particle electron cryo-microscopy revealed the principle of the assembly of four subunits, with PSEN1 in the central position, its amino terminal fragment (NTF) wrapped by PEN-2, and its carboxyl terminal fragment (CTF) interacting with APH-1. NCT’s unique TM binds to APH-1, and its extracellular domain binds to PEN-2. TM6 and TM7 in PSEN1 are located on the convex side of the TM horseshoe shape and contain catalytic aspartate residues. This structure provides an important framework for understanding the function of γ-secretase (Sun et al., 2015). The complex of γ-secretase with the Notch fragment indicates that three transmembrane domains of PSEN1 surround the transmembrane helix of Notch, and PSEN1 undergoes a significant conformational rearrangement when binding to its substrate. These features reveal structural changes in γ-secretas during substrate recruitment (Yang et al., 2019). Post-translational modifications can further regulate the matured γ-secretase. The phosphorylation of PSEN1, PSEN2 and NCT regulate functions of γ-secretase complex, including the proteolytic processing, γ-secretase activity (Fluhrer et al., 2004; Walter et al., 1999; Kuo et al., 2008), stability (Lau et al., 2002) and subcellular localization (Sannerud et al., 2016). The assembly and activation of the four subunits play an important role in the function of γ-secretase.

γ-secretase complex is ubiquitous in all tissues, the presence of all γ-secretase subunits does not guarantee active complex formation, many evidences suggest that two pools of γ-secretase exist: the long half-life of assembling γ-secretase complex and the short half-life of monomeric subunits, and only a small fraction of γ-secretase is catalytically active (Beher et al., 2003; Lai et al., 2003). A broad range of γ-secretase substrates has been identified, suggesting that additional events and cofactors composition are required to enhance the activity of γ-secretase and substrate specificity (Placanica et al., 2010). Although γ-secretase can cleave a variety of protein substrates, when cells are in different environments and receive different signals, γ-secretase may selectively hydrolyze one or a class of substrates to ensure the normal execution of cell functions. γ-secretase subunits are localized in nearly all endomembrane system compartments, including the endoplasmic reticulum (Kaether et al., 2004), lysosomal membrane (Houser et al., 2023), pre- and post-Golgi compartments (Annaert et al., 1999), phagosome (Jutras et al., 2005), plasma membrane and endosome (Vetrivel et al., 2004). This suggests that in different cellular domains γ-secretase binds to different substrates and plays different roles. Thus, the activity of γ-secretase may be regulated by a variety of mechanisms. For instance, APP is processed by intracellular γ-secretase, while Notch, which acts on the plasma membrane, is processed on the cell surface (Tarassishin et al., 2004). Furthermore, lipid composition also impacts substrate processing, the cholesterol-rich membranes are the major site of Aβ production (Wahrle et al., 2002; Marquer et al., 2014), and γ-secretase partitioning into lipid bilayers remodels membrane components that create a suitable microenvironment for substrate recognition and activity (Barros et al., 2020). Only correct subunit assembly can enable γ-secretase to recognize specific substrates under specific environment and conditions, thus being actived and performing the correct cell biological functions.

## 3 γ-secretase in Alzheimer’s disease

Over a 100 distinct type I integral membrane proteins have been identified as γ-secretase substrates to date, and more are being continuously discovered **(Hemming et al., 2008; Haapasalo and Kovacs, 2011). γ-secretase was originally characterized based on its proteolytic function in cleaving the APP, a process that generates Aβ—a key pathological component in the formation of senile plaques associated with AD. Consequently, γ-secretase is widely regarded as a central player in the pathogenesis of AD. Beyond APP, an expanding repertoire of γ-secretase substrates has been implicated in the molecular mechanisms underlying AD development. In the following sections, we will systematically examine those proteins that demonstrate significant associations with the onset and progression of AD, aiming to elucidate the multifaceted roles of γ-secretase in disease pathology and to lay a foundation for the development of targeted therapeutic interventions.**

### 3.1 Amyloid precursor protein (APP)

**FIGURE 2:**
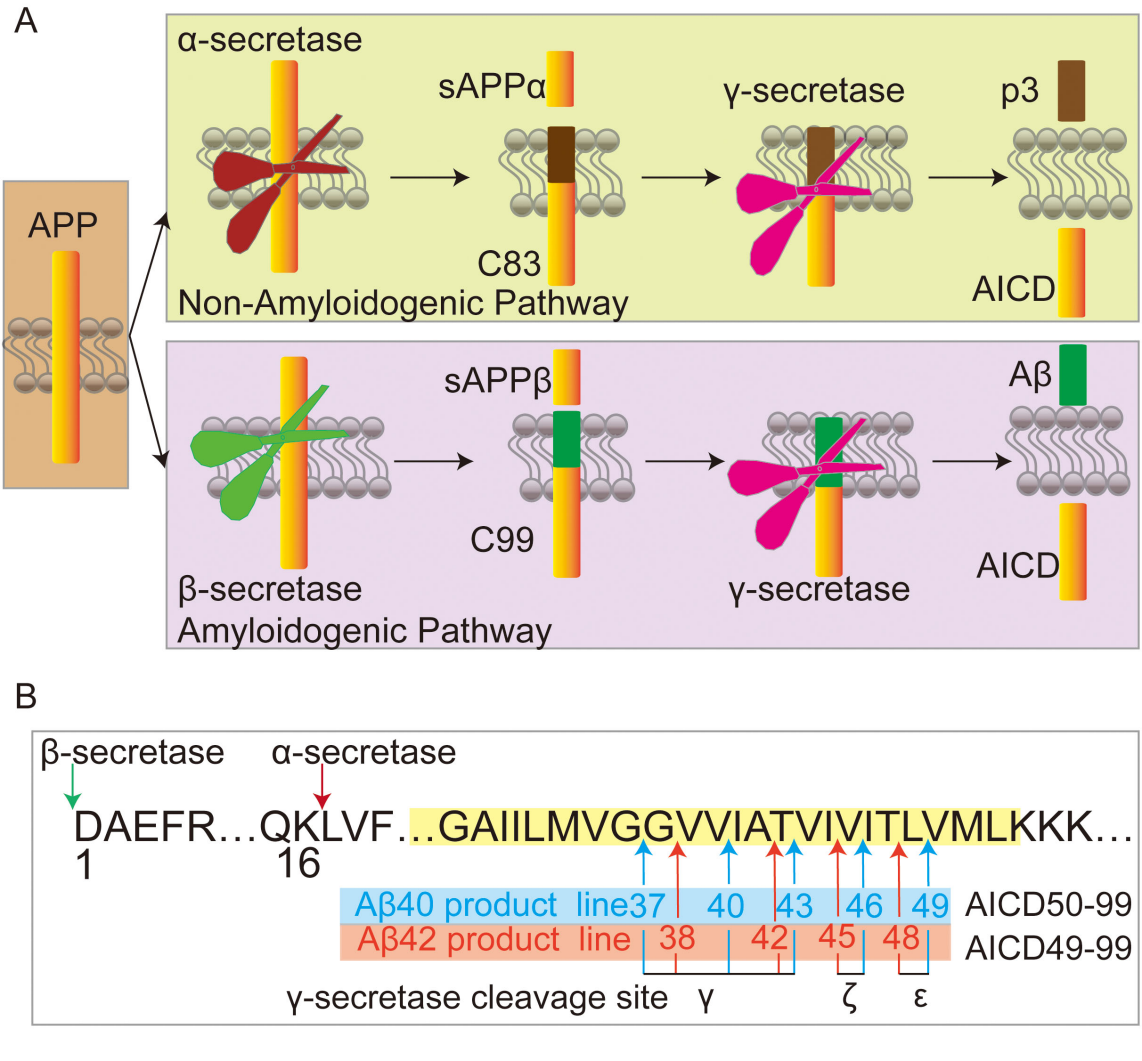
Amyloid precursor protein (APP) processing. **(A)** In the non-amyloid and amyloid pathways, APP undergoes cleavage by α-secretase or β-secretase, respectively, releasing sAPPα and C83 in the non-amyloid pathway, or sAPPβ and C99 in the amyloid pathways. Subsequently, γ-secretase cleaves C83 into p3 and APP intracellular domain (AICD), while C99 is processed into Aβ and AICD. **(B)** Following β-secretase mediated cleavage of APP (where the sequence numbering of Aβ stars at 1), C99 is further cleaved by γ-secretase at ε-sites to generate Aβ49 and AICD50-99, or Aβ48 and AICD49-99. These peptides are subsequently processed through additional cleavages at ζ and γ sites, resulting in the sequential formation of Aβ46→43→40→37 from Aβ49, and Aβ48→45→42→38 from Aβ48. Arrows indicate cleavage sites, and membranes are depicted in yellow.

Alzheimer’s disease was first officially described by Alois Alzheimer in 1906, which is the most common form of dementia (Small and Cappai, 2006). The excessively aggregates amyloid plaques in the brain majorly contribute to dysfunction and degeneration of neurons that result in AD (Zhang et al., 2012). Although the etiology of Alzheimer’s disease is still the subject of considerable debate, the “amyloid-cascade hypothesis” has remained the prevailing theory over the years (Small and Cappai, 2006; Soria Lopez et al., 2019). This hypothesis suggests that amyloid plaques in the brains of AD patients consisted of fibrils formed by Aβ. The cleavage by γ-secretase requires shedding of the substrate’s extracellular domains by the other secretases. In the Non-Amyloidogenic pathway, APP is cleaved by α-secretase to generate sAPPα and the membrane-associated 83 amino acid C-terminal fragment APP-CTF (C83), γ-secretase further cleaves C83 to produce p3 and AICD (Selkoe, 2001; Figure 2A). Alternatively, in the amyloidogenic pathway, APP is first processed by β-secretase and produces the secreted sAPP-β and APP–C-terminal 99-residue fragment (C99), subsequent γ-Secretase mediated cleavage of C99 at the γ-, ζ-, and ε-sites close to the cytosolic end of TMD to generate APP intracellular domain (AICD) and the extracellular secretion of Aβ peptides (Moser et al., 2024). The cleavage of C99 by γ-secretase results in a variety of peptides (from large Aβ49 peptides to smaller ones with 37 residues), Aβ peptides are cleaved mainly by tripeptide trimming or tetrapeptide trimming via the Aβ40 product line (Aβ49→46→43→40→37) or the Aβ42 product line (Aβ48→45→42→38) (Hur, 2022; Takami et al., 2009; Wang et al., 1996; Figure 2B).

Approximately 25 mutations in APP are associated with the occurrence of AD, many of which are located in the area of TMD (Devkota et al., 2021). Although there have been numerous debates on whether immune activity is advantageous or detrimental to the progression of AD pathology, it has long been that immune activity is closely related to the pathophysiology of AD (Frost et al., 2019).

### 3.2 Other GS substrate proteins related to AD

**FIGURE 3:**
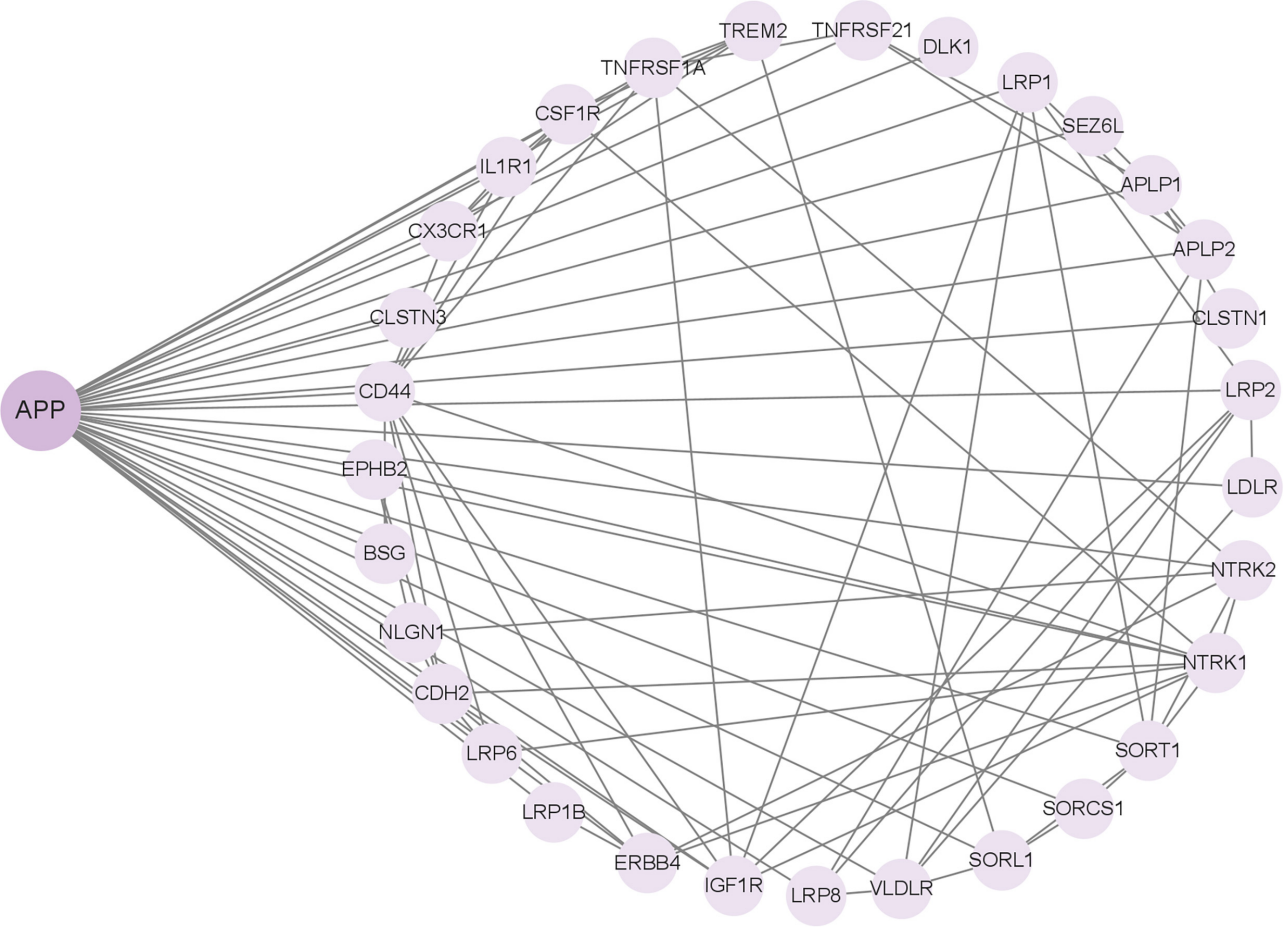
GSs substrate associated with amyloid precursor protein (APP).

In addition to APP, γ-Secretase has more than 140 substrates, all of which are type 1 transmembrane proteins. Among them, many substrates have been reported to be related to the APP. For instance, the low-density lipoprotein receptor-related protein (LRP) directly interacts with the PS1 subunit of γ-secretase and competes with APP for access to the enzyme’s cleavage site. Overexpression of the C-terminal fragment of LRP decreases the production of Aβ peptides derived from APP and suppresses the signaling activity of AICD (Lleó et al., 2005). These findings indicate that LRP functions as a competitive substrate, modulating the cleavage of APP by γ-secretase through occupancy of the enzyme’s active site. Based on the important role of APP in AD, the research on these substrates may be more conducive to our study of the molecular mechanism of AD pathogenesis. We input the 149 substrate proteins of γ-Secretase (Güner and Lichtenthaler, 2020) into the STRING website (STRING: functional protein association networks) to screen the APP-related proteins centered on this APP (Figure 3). The screened proteins were analyzed and compared with AD in the Human Disease Database Retrieval (MalaCards) and the Disgenet database. These proteins are summarized in Table 1.

**TABLE 1:** γ-secretase’s substrates associated with Alzheimer’s disease (AD).

Protein name	GDAS (MalaCards)	GDAS (disgenet)	Functions in AD
Alcadein α (Calsyntenin-1) (Hata et al., 2009)	22.12	0.2	Binding to kinesin-1 motors to mediate axonal anterograde transport of certain types of vesicles. APP is transported through these vesicles (Araki et al., 2004)
Alcadein γ (Calsyntenin-3) (Hata et al., 2009)	NR	0.2	Regulate synaptic function (Uchida et al., 2013)
APLP1 (Schauenburg et al., 2018)	24.01	0.35	APLP1 plays a role in the formation and maturation of cortical synapses. (Kim et al., 1995)
APLP2 (Eggert et al., 2004)	33.56	0.3	Regulate neural development and myelination (Roisman et al., 2019)v
apoER2(LRP8) (Fuentealba et al., 2007)	20.68	0.8	Regulate synaptic plasticity and Aβ (amyloid-beta) metabolism (Kim et al., 2014)
APP (Xu et al., 2016)	567.73	1	The basis for the formation of amyloid plaques in the brains of patients with Alzheimer’s disease (De Strooper et al., 1998)
CX3CR1 (CCRL1) (Güner and Lichtenthaler, 2020)	NR	NR	CX3CL1 ICD may hold significant translational potential for neuroprotection in Alzheimer’s disease and for disorders associated with insulin resistance (Gayen et al., 2022).
CD147 (Basigin BSG) (Güner and Lichtenthaler, 2020)	14.51	NR	CD147 subunit within the gamma-secretase complex down-modulates the production of Abeta-peptides (Zhou et al., 2005).
CD44 (Lammich et al., 2002)	13.04	NR	CD44 encodes a cell surface glycoprotein that is associated with inflammation, metastasis, and inflammatory-linked neuronal damage (Pinner et al., 2017).
CSF-1R (Wei et al., 2021)	131.9	0.96	Microglia are dependent on signaling through the colony stimulating factor-1 receptor (CSF-1R/CD115) for growth and survival (Walker et al., 2017).
Delta1(DLK1) (Ikeuchi and Sisodia, 2003)	NR	NR	Regulate neuroinflammation and the functions of glial cells (Zheng and Wang, 2025).
DR6 (TNFRSF21) (Colombo et al., 2018)	NR	0.25	Regulate neuronal degeneration and neuroinflammation (Zeng et al., 2012)
EphB2 (Xu et al., 2009)	NR	NR	Regulate synaptic plasticity and the inflammatory response (Shi X. D. et al., 2016)
ErbB4 (Ni et al., 2001)	NR	NR	Regulate synaptic plasticity, neuroinflammation, as well as Aβ (amyloid-beta protein) (Chaudhury et al., 2003).
IGF-1R (McElroy et al., 2007)	13.81	0.8	The IGF 1/IRS-2 signaling pathway can regulate the activities of alpha-/beta-secretase,and inhibit the production of Aβ (amyloid-beta) (Freude et al., 2009)
IL-1R1 (Elzinga et al., 2009)	NR	0.45	Reducing the interleukin-1β receptor-1 (IL-1R1) negative regulator TOM1 affected microglia activity, increased amyloid-beta levels (Martini et al., 2019).
LDLR (Kim et al., 2018)	19.79	0.85	Regulate the cholesterol metabolism in the brain (Abisambra et al., 2010)
LRP1 (Zurhove et al., 2008)	44.34	0.8	LRP1 is involved in efflux of amyloid beta-peptide (1-42) (Sultana et al., 2010)
LRP1b (Liu et al., 2007)	NR	NR	Regulate neurodegeneration (Benoit et al., 2013)
LRP6 (Mi and Johnson, 2007)	21.9	0.35	Regulate the function and stability of synapses (Buechler and Salinas, 2018)
LRP2 (Zou et al., 2004)	NR	0.35	LRP2 deficiency mice at the mediating BBB leads to neurodegeneration (Dietrich et al., 2014).
N-cadherin (CDH2) (Marambaud et al., 2003)	NR	NR	The cleaved levels of N-cadherin were increased in homogenates of postmortem brain from AD patients compared with that in non-AD patients. (Choi et al., 2020)
Neuregulin-1 (NLGN1) (Malvankar and Wolfe, 2025)	NR	NR	Neuro protective effect (Sun et al., 2020)
SEZ6L (Pigoni et al., 2016)	NR	NR	SEZ6L is a neuronal substrate of the AD β-secretase, and function in the nervous system (Ong-Pålsson et al., 2022)
SorCS1 (Nyborg et al., 2006)	NR	NR	Regulate synaptic formation and function (Savas et al., 2015)
SorLA (Sorl1) (Nyborg et al., 2006)	NR	0.65	Regulate the processing of amyloid precursor protein (APP), the metabolism of Aβ (amyloid-beta protein), and the intracellular transportation within neurons (Jensen et al., 2023).
Sortilin (Nyborg et al., 2006)	14.03	0.65	Participate in the regulation of the clearance of Aβ (amyloid-beta) (Andersson et al., 2016)
TkrB (NTRK2) (Tejeda et al., 2016)	NR	NR	TrkB receptor agonist blocks δ-secretase activity exhibiting promising therapeutic efficacy in 3xTg AD mouse model (Liao et al., 2021).
TNFRSF1A (TNFR1) (Chhibber-Goel et al., 2016)	NR	NR	A novel substrate of γ -secretase composed of ps1. The IL-1R1 ICD produced by γ -secretase translocates to the nucleus under the stimulation of IL-1β (Chhibber, 2013).
TREM2 (Wunderlich et al., 2013)	117.71	1	TREM2 exerts an anti-inflammatory effect in the brain (Jonsson et al., 2013).
TRKA(NTRK1) (Merilahti et al., 2017)	21.3	0.8	It mediates the signal transduction of nerve growth factor (NGF) and regulates neuronal survival, synaptic plasticity, and the metabolism of pathological proteins (Chen et al., 2008).
VLDLR (Hoe and Rebeck, 2005)	21.24	0.8	Regulate lipid metabolism (Moulton et al., 2021)

The table sequentially lists the names and species of the proteins, the gene scores related to Alzheimer’s disease (AD) from the MalaCards and Disgenet databases, as well as their roles in AD. NR, not retrieved; GDAS, gene-disease association score.

## 4 γ-secretase modulators (GSMs)

### 4.1 GSMs

**FIGURE 4:**
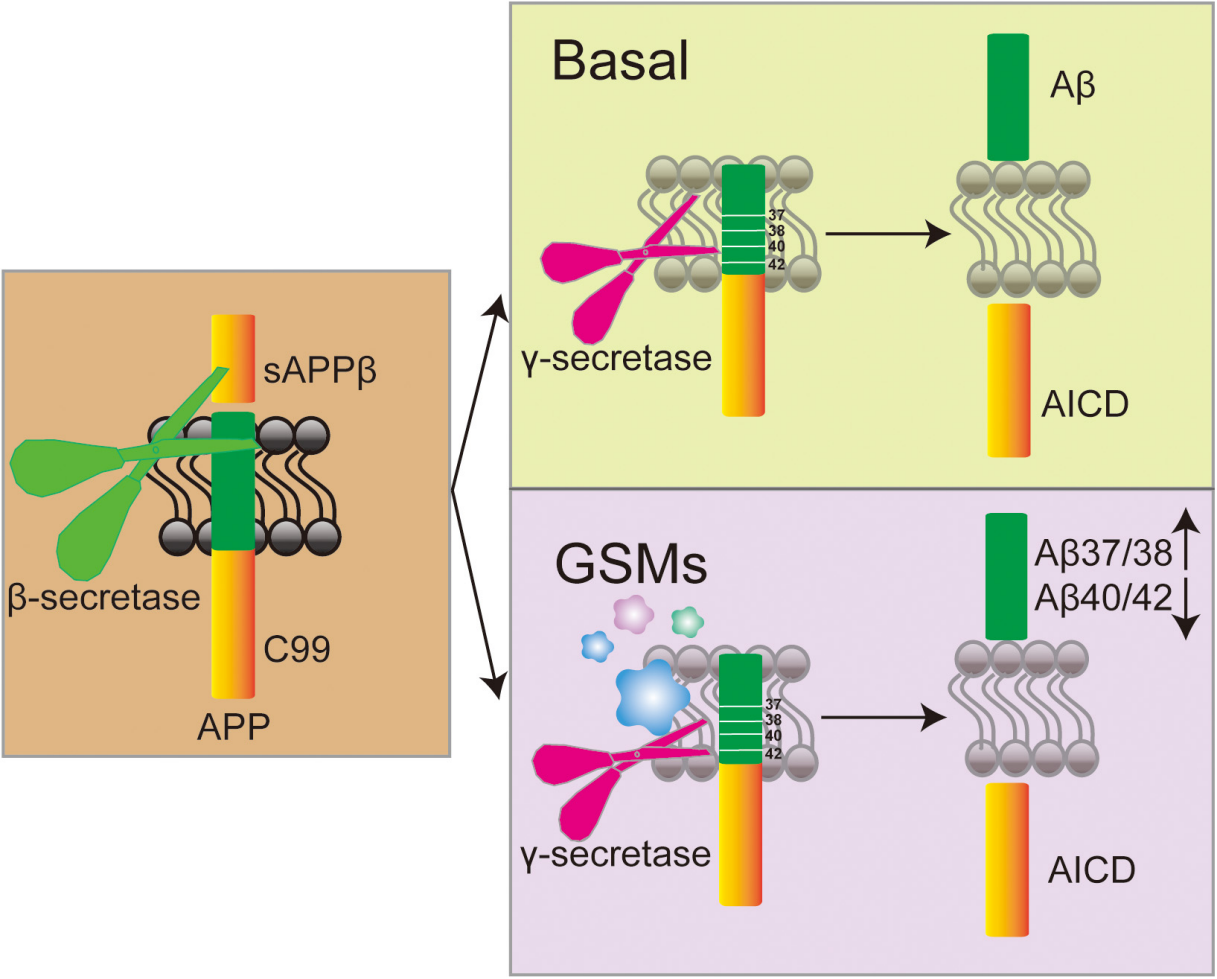
Mechanism diagram of γ-secretase modulators (GSMs) regulating γ-secretase activity and its effect on Aβ peptide generation.

γ-secretase modulators exhibit greater therapeutic potential by modulating enzyme activity to selectively decrease Aβ42 levels while preserving the normal processing of other substrates (Mekala et al., 2020; Wolfe, 2012). Cryo-electron microscopy revealed that GSMs bind to the transmembrane domain of PS1 and change the conformation of γ-secretase, thereby reducing Aβ42 production and enhancing Aβ38 generation (Yang et al., 2021; Mehra and Kepp, 2021; Figure 4). In addition, Petit et al. demonstrated that certain GSMs enhance the efficiency of substrate processing by stabilizing the interaction between γ-secretase and APP, elucidating the mechanism of their selective regulation (Petit et al., 2022). Early GSMs, such as non-steroidal anti-inflammatory drugs (NSAIDs), were ineffective due to insufficient potency and unfavorable pharmacokinetic properties. NSAIDs mainly alleviate Aβ-induced neuroinflammation by inhibiting COX-1, COX-2 and prostaglandins (Imbimbo et al., 2010). Ibuprofen, flurbiprofen, and indomethacin can regulate γ-secretase, lowering Aβ42 levels while increasing Aβ38 (Eriksen et al., 2003). However, clinical trials have not confirmed significant efficacy in patients with mild to moderate AD (Miguel-Álvarez et al., 2015). Although studies on indomethacin have shown some positive trends, the results are not convincing due to the small sample size (de Jong et al., 2008). Research indicates that NSAIDs may be effective in the early stage of the disease but ineffective or even harmful in the later stages (Hampel et al., 2020). Moreover, most NSAIDs have difficulty penetrating the blood-brain barrier (BBB), limiting their efficacy (Sastre and Gentleman, 2010), and the effects are also influenced by individual genetic backgrounds (DiBattista et al., 2016). Recently, novel GSMs, including pyridine derivatives, purine compounds, and quinazolinones, have exhibited greater potency and selectivity. The purinergic GSMs developed by Rivkin’s group substantially reduced brain Aβ42 levels in a mouse model (Rivkin et al., 2010). The incorporation of lipophilic groups has been shown to improve membrane permeability and central exposure (Naumann et al., 2013; Sekioka et al., 2020b). The newer-generation GSM BPN-15606 does not inhibit overall γ-secretase activity. Instead, it binds to the PS1 subunit of γ-secretase, allosterically altering APP cleavage to reduce Aβ42/Aβ40 production and promote the generation of more easily cleared fragments Aβ38/Aβ37 (Wagner et al., 2017). In PSAPP transgenic mice, treatment with BPN-15606 before significant plaque formation effectively reduced amyloid deposition and improved cognitive performance. However, when treatment started six months of age—after plagues were already widespread—the drug reduced Aβ pathology but did not improve existing cognitive impairments (Prikhodko et al., 2020). This suggests that once neuronal damage becomes irreversible, lowering Aβ levels alone may not be sufficient to restore cognitive function. Mobley’s team in Down syndrome mice showed that BPN-15606 significantly reduced Aβ42 and Aβ40 levels; improved nerve growth factor signaling; reduced tau hyperphosphorylation, and corrected behavioral deficits, indicating its potential to delay or prevent AD onset in individuals with Down syndrome (Chen et al., 2024). Preclinical studies also demonstrated favorable pharmacokinetics and safety profiles supporting its advancement into clinical trials (Wagner et al., 2017; Gunnar et al., 2023). Although the new generation of GSMs possesses theoretical safety advantages based on their mechanism of action, practical challenges remain for these modulators. Firstly, the substrate diversity of γ-secretase may result in off-target effects, necessitating the development of more precise modulators. Secondly, the activity of γ-secretase in the brains of AD patients varies with disease progression, potentially influencing drug efficacy (Nobuto et al., 2013). Future research should focus on structure-based drug design, combining cryo-electron microscopy and computational chemistry to optimize binding sites. Developing multi-targeted modulators for Aβ and tau pathologies is also crucial (Gunnar et al., 2023; Joanna and Yue-Ming, 2022; Ji-Yeun, 2022). The existing GSMs are summarized in Table 2.

**TABLE 2:** The existing γ-secretase modulators (GSMs).

Name	Mechanism of drug action	Phase	Status	Side effect
Ibuprofen	modulate γ-secretase to lower the production of Aβ42 and concomitantly increase Aβ38 without affecting the total amount of Aβ (Weggen et al., 2001)	III	Completed	Weak efficacy or insufficient targeting (Ling et al., 2015)
Indomethacin	Selectively reducing the levels of Aβ42 while simultaneously increasing the production of soluble Aβ38 without affecting Notch signaling.	III	Completed	Damage to the gastric mucosa, ulceration, and bleeding (Sava, 2021)
Sulindac sulfide	Selective inhibition of Aβ42 production and increase of Aβ38 and Aβ37 fragments (Barrett et al., 2011)	Preclinical	Suspended	Gastrointestinal pain, Dizziness/headache (Brogden et al., 1978)
R-flurbiprofen	Selective binding to the active site of γ-secretase modulates its cleavage preference, consequently diminishing the production of Aβ42. (Höttecke, 2010)	III	Terminated	Low potency and poor CNS penetration (Green et al., 2009)
CHF-5074	CHF-5074 binds to the intracellular domain of APP, altering the cleavage site of γ-secretase and preferentially reducing Aβ42 production (Branca et al., 2014)	II	Completed	Mild diarrhea (Amirrad et al., 2017)
EVP-0962	It decreases the production of Aβ42 peptide and increases the proportion of short-chain Aβ38, thereby decreasing amyloid plaque formation (Bulic et al., 2011)	II	Completed	It was discontinued after a phase II clinical trial, and the results are not reported (Hur, 2022)
E2012	Targeting the hydrophobic pocket within the transmembrane domain of PS1-NTF (Luo and Li, 2022)	I	Terminated	Affecting cholesterol metabolism, leading to lenticular opacity (Nakano-Ito et al., 2014)
E2212	Reducing Aβ42/40 peptides while increasing soluble Aβ37/Aβ38 (Maia and Sousa, 2019)	I	Completed	Diarrhea (Yu et al., 2014)
PF-06648671	Reductions of Aβ42 and Aβ40, together with increases in Aβ37 in healthy volunteers (Ahn et al., 2020)	III	Terminated	No major side effects were reported.
BMS-932481	increase in Aβ37 and reduce of Aβ42 CSF levels in healthy volunteers (Boy et al., 2019)	II	Terminated	Liver injury, alanine aminotransferase (ALT) elevated (Zhuo et al., 2023)
NGP555	Binding of the interacting regions of Pen-2, Nct, and TM loops 3-4 to regulate Aβ peptide generation (Ioppolo et al., 2021)	I	Completed	Headache, nausea, and dizziness, liver enzyme levels were elevated (Rynearson et al., 2021)
BIIB042	Binding to PSEN and induces γ-secretase conformational change, decrease Aβ42, increase Aβ38 and have no effect on Aβ40 levels (Scannevin et al., 2016).	II	Suspended	No relevant studies
RO7185876	Altering the cleavage site of APP, reduces Aβ40 production and increases Aβ37/Aβ38 (Ratni et al., 2020)	Preclinical	Unclear	No relevant studies
RG6289	Decrease Aβ42/Aβ40 and elevations in Aβ38 and Aβ37 (De Strooper and Karran, 2024)	II	Active	Mild headache, gastrointestinal discomfort
BPN-15606	Lowering Aβ42 accumulation	Preclinical	Active	High Dose toxicity in animal (Wagner et al., 2017)
UCSD-776890	Potent *in vitro* and *in vivo* Aβ42 inhibition;	Preclinical	Active.	No sign of cardiac toxicity and mutagenicity (Rynearson et al., 2021)
RO7019009	Increasing Aβ37/38, reducing Aβ40/42/43, has greater therapeutic potential for patients carrying a single PS1 L166P allele. (Trambauer et al., 2025)	Preclinical	Active	No reports
ACD680	Decrease Aβ42/Aβ40 and elevations in Aβ38/Aβ37 (Nordvall et al., 2025)	Preclinical	Active	No reports
UCSD-779690	Potent Aβ42 inhibition	Preclinical	Unclear	No sign of cardiac toxicity and mutagenicity (Rynearson et al., 2021)
Cpd133	Inhibition of Aβ42 and solubility	Preclinical	Unclear	Poor ADME (Rynearson et al., 2016)
Cpd134	Decreasing Aβ42 in cell-based assay	Preclinical	Unclear	Cardiac toxicity potential (Rynearson et al., 2020)
Cpd135	Inhibition of Aβ42	Preclinical	Unclear	CYP3A4 inhibition (Shi J. et al., 2016)
Cpd136	Decreasing Aβ42	Preclinical	Unclear	Limited *in vivo* activity and poor drug-like properties (Wu et al., 2016)
FRM-36143	Decrease Aβ42 and elevations in Aβ38 and Aβ37 (Bursavich et al., 2017; Blain et al., 2016)	Preclinical	Unclear	No reports
FRM-024	Improved Aβ42 reduction (Bursavich et al., 2021)	Preclinical	Unclear	No reports
Cpd142	Improved Aβ42 reduction (Zhao et al., 2017)	Preclinical	Unclear	No reports
Cpd143	Improved Aβ42 reduction (Fischer et al., 2015)	Preclinical	Unclear	Off-target effects
PF-06442609	Potent Aβ42 lowering activity (Pettersson et al., 2015)	Preclinical	Unclear	No reports
Cpd145	Aβ42 reduction (Pettersson et al., 2017)	Preclinical	Unclear	No reports
BI-1408	Aβ42 reduction (Gerlach et al., 2018)	Preclinical	Unclear	No reports
Cpd147	Lowering Aβ42 accumulationinvitroandinvivo (Takai et al., 2015)	Preclinical	Unclear	No reports
Cpd149	Effectively inhibiting Ab42 (Murakami et al., 2016)	Preclinical	Unclear	No reports
RO7101556	Potent *in vitro* and *in vivo* activity (Rodríguez Sarmiento et al., 2020)	Preclinical	Unclear	No reports
Cpd152	Lack of *in vivo* efficacy (Sekioka et al., 2018)	Preclinical	Unclear	No reports
Cpd153	Efficacious Ab42 lowering (Sekioka et al., 2020b)	Preclinical	Unclear	No reports
Cpd154	Potent Ab42 inhibition *in vitro* and *in vivo* (Sekioka et al., 2020a)	Preclinical	Unclear	No reports
Cpd165	Potent Ab42 *in vitro* and *in vivo* lowering (Bischoff et al., 2019)	Preclinical	Unclear	No reports
D-Pinitol	Altering APP processing pathways reduces Aβ42 production while avoiding interference with Notch signaling (López-Sánchez et al., 2018)	Preclinical	Unclear	No reports
2,5-disubstituted 2-benzylidene hexanoic acid derivatives	act as dual γ-secretase/PPARγ modulators in the low micromolar range (Hieke et al., 2011)	Preclinical	Unclear	No reports
1,2,3-C-aryl-triazoles	Good modulation of γ-secretase activity, excellent pharmacokinetics, and reduced central Aβ42 levels in Sprague-Dawley rats (Fischer et al., 2011)	Preclinical	Unclear	No reports
Compound 10 h	Decreasing the level of Aβ42 in plasma, CSF, and brain, with little effect on the level of Aβ40 (Hawkins et al., 2011)	Preclinical	Unclear	No reports
Iminoheterocycles	Lowering Aβ42 levels in various *in vivo* models (Caldwell et al., 2010)	Preclinical	Unclear	No reports

### 4.2 γ-secretase modulatory proteins (GSMPs)

The aggregation of Aβ-monomers forms the amyloid plaques observed in Alzheimer patients’ brains, which is the key factor in AD (Selkoe, 2013). Increasing evidence has suggested that γ-secretase and its associated proteins could participate in regulating AD, but direct inhibition of γ-secretase has no significant effect on reducing Aβ accumulation and improving cognition in AD’s patients (Shi and Holtzman, 2018). γ-secretase has been a key target for drugs as it facilitates the final cleavages in the production of Aβ. Due to severe adverse effects in clinical trials, GSIs have been discontinued. GSMPs are more secure. It has high efficiency and good safety. Targeting GSMPs can selectively regulate the APP pathway and has tremendous applicability prospects in the treatment of AD. Nowadays, several GSMPs that are closely related to the APP signaling pathway, such as GSAP (Kim et al., 2024; Jin et al., 2022), IFITM3 (Hur, 2021), transmembrane trafficking protein, 21-KD (TMP21) (Ílgün and Çakir, 2025), and stress-associated endoplasmic reticulum protein 1 (SERP1) (Jung et al., 2020). We review the results of a Pubmed search for the keywords “gamma secretase AND (γ-secretase modulatory proteins OR GSMP)” and review recent advances in GSMPs.

#### 4.2.1 γ-secretase activating protein (GSAP)

Lots of γ-secretase interacting proteins have been identified through LCMS analysis. The GSAP was identified as a γ-secretase-interacting partner (Teranishi et al., 2010; Frykman et al., 2012), it could form a complex with γ-secretase and APP-CTF, and an SNP in GSAP has been discovered to be associated with Alzheimer’s disease, giving genetic evidence that links GSAP to AD susceptibility (He et al., 2010). Studies have shown that the inhibition of GSAP can reduce Aβ production without affecting the other key γ-secretase substrates (Kim et al., 2024). GSAP does not interact with Notch, nor does it affect its cleavage (He et al., 2010; Wong et al., 2019). Knockdown of GSAP could reduce Aβ burden and plaque development in the AD model transgenic mice (Kim et al., 2024; He et al., 2010). GSAP specifically promotes γ-secretase–mediated cleavage of APP, the 16-kDa C-terminal fragment of GSAP (GSAP-16K) exerts dual modulation on γ-secretase cleavage: GSAP-16K in dilute phase increases C99 cleavage toward preferred production of Aβ42, but GSAP-16K condensates reduce APP-C99 cleavage through substrate sequestration (Jin et al., 2022). Imatinib is a commonly used anti-cancer drug in the clinic, multiple evidence indicates that imatinib inhibits Aβ but has no effect on Notch signaling (He et al., 2010; Netzer et al., 2003). In AD mouse models, imatinib prevents GSAP from interacting with APP-CTF, thereby reducing Aβ levels and tau phosphorylation (Chu et al., 2014). However, due to the extremely poor BBB penetration ability of imatinib (Brahmachari et al., 2017), it has not yet been applied in Phase II and Phase III clinical trials. Administration of the dimerization of Lep9R3LC (diLep9R3LC) peptide and GSAP siRNA complexes in AD mice can reduce GSAP in the cortex/hippocampus, inhibit Aβ accumulation, reduce tau hyperphosphorylation, and improve cognitive function in AD mice (Kim et al., 2024). In neurons, GSAP interacts with the Fe65–APP complex to modulate APP phosphorylation and its trafficking/partitioning (Xu et al., 2021). Reducing GSAP levels diminishes the partitioning of APP-CTF into lipid rafts, specifically at the mitochondria-associated membrane (MAM), and also decreases γ-secretase activity involved in Aβ production, and decreasing GSAP expression mitigates pathological effects linked to AD (Xu et al., 2021). In addition to contributing to amyloid formation in the brain, GSAP can also promote end-organ dysfunction after bacterial pneumonia. Infection with Pseudomonas aeruginosa caused arterial hypoxemia in wild-type rats, but the integrity of the alveolar-capillary barrier was preserved in GSAP knockout rats. Additionally, infection enhanced myocardial infarction after ischemia-reperfusion injury, and this enhancement was eliminated in the GSAP knockout rats (Gwin et al., 2023). This result sheds light on the role of GSAP in innate immunity and highlights the contribution of GSAP to end-organ dysfunction during infection. However, even if GSAP can serve as an amyloid-beta-lowering therapeutic target without affecting other key functions of γ-secretase, but whether it also acts on other substrates and whether its regulation causes other side effects remains to be investigated.

#### 4.2.2 Interferon-induced transmembrane protein 3 (IFITM3)

Interferon-induced transmembrane protein 3 is one of the members of the IFITMs family, as interferon-induced genes have been found in human neuroblastoma cells, it is an innate immune response protein involved in preventing the entry of viruses into host cells (Amini-Bavil-Olyaee et al., 2013; Bailey et al., 2014; Kehs et al., 2025). The Aβ peptides have antimicrobial and antiviral activities as part of the innate immune response in the brain (Amini-Bavil-Olyaee et al., 2013; Bailey et al., 2014). Hur et al. (2020) performed photo-crosslinking tests using the γ-secretase modulator E2012-BPyne and found that IFITM3 is a γ-secretase interaction protein. By interacting with neighboring IFITMs or other transmembrane proteins, IFITMs inhibit the formation of viral fusion pores and reduce the fluidity of the host membrane, this prevents viral infection (Yao and Yan, 2020). In the context of AD, IFITM3 was significantly reduced in the PSEN1 and PSEN2 double-knockout mice, and similarly, IFITM3 RNAi also reduced γ-secretase activity. Furthermore, IFITM3 directly binds to γ-secretase near the catalytic site and reduces γ-secretase activity for Aβ40 and Aβ42 (Hur et al., 2020). The expression of IFITM3 is significantly higher in patients with Alzheimer’s disease in the temporal cortex, knockout of IFITM3 in AD mouse significantly reduces plaque deposition (Hur et al., 2020). Other studies have shown that the expression of IFITM3 could be upregulated by the human herpes virus 6B, hepatitis C virus and cytokines interferon-γ (IFNγ), IFNα, IL-6, and IL-1β all significantly induce (Gómez-Herranz et al., 2023; Yánez et al., 2020; Jiménez-Munguía et al., 2022), and when infected COVID- 19, young mice showed higher IFITM3 responses and interferon-induced chemokines than older mice (Subramaniam et al., 2024). As an immune response protein, IFITM3 plays an important role in AD and other immune-related diseases. It has been confirmed that IFITM3 can interact with γ-secretase in AD, but whether γ-secretase is also involved in other immune diseases with IFITM3 remains to be confirmed.

#### 4.2.3 Transmembrane trafficking protein, 21-KD (TMP21)

Transmembrane trafficking protein, 21-KD, also known as TMED10, is expressed in most brain regions, with higher expression in neuronal cells (Vetrivel et al., 2008). Transgenic mice with neuron-specific increases in TMP21 expression exhibit postnatal growth retardation and severe neurological issues like tremors, seizures, ataxia, uncoordinated movements, and premature death, complete deletion of TMP21 results in embryonic lethality at very early stages (Denzel et al., 2000; Gong et al., 2011). TMP21 is co-localized with γ-secretase, its expression is decreased in AD, which is consistent with the previous study that Aβ expression is increased when TMP21 is knocked down (Vetrivel et al., 2008; Pardossi-Piquard et al., 2009; Chen et al., 2006). Importantly, TMP21 binds to γ-secretase and specifically modulates APP cleavage at the γ-site, however, it has no effect on Notch cleavage at the ε-site (Vetrivel et al., 2008; Chen et al., 2006; Bromley-Brits and Song, 2012). Moreover, TMP21 reduction impairs APP’s bidirectional transport in the ER/Golgi, which increases the amount of APP that undergoes amyloidogenic cleavage in endocytic compartments and sAPP, Aβ40, and Aβ42 secretion (Vetrivel et al., 2007). Reducing TMP21 can increase GSK3β activity (Zhang et al., 2019), thereby promoting NF-κB-mediated β-site amyloid precursor protein cleaving enzyme 1 (BACE1) expression and activity, which further promotes APP processing and Aβ generation (Ly et al., 2013). A study showed that TMP21 could interact with the murine cytomegalovirus immunoevasin gp40 to facilitate virus immune escape (Ramnarayan et al., 2018; Stützer et al., 2013; Xu et al., 2023; Araki et al., 2004; Honda et al., 2024), so does TM21 also defend against foreign invasion in AD like IFITM3? This is also one of the directions that can be explored in the future.

#### 4.2.4 Stress-associated endoplasmic reticulum protein 1 (SERP1)

Through a genome-wide screen for regulators of γ-secretase activity, researchers identified that SERP1 promotes Aβ production in cells undergoing endoplasmic reticulum (ER) stress. The carboxyl-terminal domain of SERP1 interacts with the APH1A/NCT subcomplex of γ-secretase. Under ER stress conditions, SERP1 selectively recruits APP into the γ-secretase and enhances the subcellular localization of this complex within lipid rafts, leading to increased Aβ generation. Moreover, in cells exposed to high glucose levels and in diabetic AD model mice, elevated levels of SERP1, enhanced γ-secretase assembly, and increased Aβ production were observed (Jung et al., 2020). APH1A and NCT play a crucial role in stabilization, maturation and substrate recognition of the γ-secretase complex. The binding of SERP1 to this subcomplex may enhance its enzymatic activity by stabilizing the conformation of the complex or facilitating the entry of substrates into the active site. Although SERP1 has been shown to interact with γ-secretase, no atomic-resolution cryo-EM structures of SERP1 in complex with either the full γ-secretase complex or its APH1A/NCT subcomplex have been reported to date. Cryo-EM technology holds the potential to elucidate the molecular details of the SERP1–γ-secretase interaction at the atomic level, which is critical not only for understanding the unique mechanism of SERP1 as a GSMP, but also for guiding the rational design of therapeutics targeting this interaction.

### 4.3 The other GSMPs

#### 4.3.1 Hif-1α

Hypoxia-inducible factor 1α (Hif-1α) serves as a key transcription factor in the cellular adaptation to hypoxic conditions. It plays critical roles in processes such as angiogenesis, metabolism (Masoud and Li, 2015), embryogenesis, and the development and progression of tumors (Peng and Liu, 2015). Numerous studies have shown that Hif-1α can enhance Notch signaling by binding to and stabilizing the intracellular domain of Notch (NICD), thereby activating downstream Notch target genes. Under hypoxic conditions, the interaction between Hif-1α and Notch is essential for maintaining the undifferentiated state of cells (Gustafsson et al., 2005). To counteract the negative feedback effects of Notch signaling in cancer stem cells, Hif-1α can bind to the promoter region of Hes1, a gene targeted by Notch signaling (Wang et al., 2011). In *Drosophila* models, Hif-1α has been shown to activate Notch signaling independently of ligand interactions, promoting the survival of *Drosophila* blood cells (Mukherjee et al., 2011). Additionally, research has demonstrated that hypoxia leads to an increase in Aph1a gene expression, which contributes to the upregulation of γ-secretase activity. This effect is mediated through a Hif-1α response element located within the Aph1a gene promoter (Wang et al., 2006). In breast cancer, Hif-1α no longer performs its canonical role as a transcription factor under hypoxic conditions, but instead interacts directly with γ-secretase regulates its activity for Notch cleavage, and enhances cancer cell migration and metastasis (Villa et al., 2014). Microglia-specific BACE-1 deletion enhances autophagolysosome function and Aβ-induced metabolic reprogramming via PI3K-mTOR-HIF-1α signaling, promoting Aβ degradation (Singh et al., 2022). This demonstrates that Hif-1α plays a significant role in AD; however, the molecular mechanism by which Hif-1α and γ-secretase jointly regulate AD requires further elucidation.

#### 4.3.2 CD 147

Coimmunoprecipitation investigations indicated that CD147, a glycoprotein, also referred to as basigin or EMMPRIN (Gabison et al., 2009). Inhibition of CD147 was found to enhance the production of Aβ-peptides, suggesting its function as a regulatory component of γ-secretase in Aβ generation (Zhou et al., 2005). Additionally, CD147 regulates matrix metalloproteinases and is expressed across various human tissues, contributing to both extracellular matrix degradation and fibrosis. This makes it a promising target for cancer therapy via interactions involving cell-matrix and cell-cell connections (Huang et al., 2023; Gao et al., 2016). CD147 undergoes intramembrane cleavage by γ-secretase at lysine 231, leading to the release of its intracellular domains (ICD). The CD147ICD subsequently relocates to the nucleus, where it activates Notch signaling by binding to Notch promoters (Yong et al., 2019). Elevated levels of nuclear CD147ICD correlate with poorer prognoses in human hepatocellular carcinoma (HCC), with patients exhibiting high CD147ICD expression showing significantly reduced overall survival rates compared to those with low CD147ICD expression (Yong et al., 2019). Depletion of CD147 using RNAi increases Aβ-peptide production without altering γ-secretase or APP substrates. Understanding the molecular mechanisms underlying the interaction between CD147 and γ-secretase could pave the way for developing innovative therapies for Alzheimer’s disease (Zhou et al., 2005).

## 5 Concluding remarks and future challenges

The γ-secretase complex is universally present in all cells and tissues, and its ability to cleave multiple type I membrane proteins can be likened to “molecular scissors “(Wong et al., 2020). Initially characterized as a proteolytic activity responsible for the cleavage of APP to generate Aβ, γ-secretase has been extensively studied in the context of FAD due to missense mutations in its subunits (Jarrett et al., 1993). The deposition of Aβ peptide plaques in the cerebral cortex represents a hallmark feature of AD patients (Dominguez-Gortaire et al., 2025). The generation of Aβ peptides is not only dependent on γ-secretase activity but is also critically regulated by β-secretase. Following the cleavage of APP by β-secretase at the β-site, a membrane-bound C99 intermediate is formed. This C99 fragment subsequently undergoes further cleavage by γ-secretase to produce Aβ peptides. Thus, β-secretase plays a pivotal role in controlling the production of Aβ (Cervellati et al., 2021). In AD, β-secretase expression and activity are significantly increased, contributing to excessive Aβ production (Vassar, 2012). β-secretase inhibitors (BSIs) could reduce Aβ40 and Aβ42 levels effectively (Eketjäll et al., 2016). However, β-secretase has dual roles in Aβ, both in its production and degradation. When β-secretase is highly expressed, low-dose inhibitors may instead lead to an increase in Aβ42 and Aβ40 levels, as the Aβ-degrading activity of β-secretase is preferentially weakened (Ulku et al., 2024). Several BSIs have advanced to phase III clinical trials, but none have shown clear clinical benefits. For example, Verubecestat caused reversible cognitive decline (Vassar, 2012); Atabecestat led to dose-dependent liver enzyme elevation (Pleen and Townley, 2022; Evin et al., 2011); Lanabecestat was linked to psychiatric events, weight loss, and skin depigmentation (Patel et al., 2022); and Elenbecestat resulted in dizziness, nightmares, elevated liver enzymes, and hippocampal atrophy Elenbecestat (Patel et al., 2022). Despite the setbacks encountered in phase III clinical trials, the involvement of β-secretase in AD pathology remains widely acknowledged. The complexity of AD pathogenesis and the potential off-target effects of BSIs are considered critical contributors to the development of adverse drug reactions.

After β-secretase cleaves APP, γ-secretase subsequently cleaves the resulting C99 fragment to generate Aβ peptides. Recent studies have shown that the formation of Aβ peptide plaques may serve as a defense mechanism against viral attacks (Hur et al., 2020). Thus, it can be hypothesized that under normal physiological conditions, plaques fulfilling their function are subsequently cleared. However, when this clearance process is impaired, it leads to the development of AD. With an increasing number of identified γ-secretase substrates, it has become evident that, in addition to APP, several substrates play critical roles in AD progression (Yin et al., 2025). The limited clinical progress achieved through targeting APP alone suggests that the interplay between these substrates and APP warrants further investigation. Due to the broad substrate specificity of γ-secretase, GSIs inhibit the processing of multiple substrates, leading to significant side effects (Chen et al., 2023). Particularly the Notch pathway, which plays a critical role in essential cellular processes such as stem cell maintenance and proliferation, cell fate determination, and differentiation (Kovall et al., 2017; Kopan and Ilagan, 2009). Inhibition of Notch has been associated with gastrointestinal disorders (Hur, 2022; Abdallah, 2024); lymphopenia and an elevated risk of infection (Doody et al., 2013; Wong et al., 2004), cognitive decline (Doody et al., 2013), and an increased incidence of skin cancer (Uddin et al., 2020). Recent evidence indicates that γ-secretase inhibitors can impair epithelial cell function, leading to colitis in mice (Erkert et al., 2025). Compared to GSIs, GSMs offer more precise modulation of the γ-secretase cleavage site. They shift the cleavage position within the APP transmembrane region, leading to a decrease in Aβ42 levels and a relative increase in Aβ37 and Aβ38 (Figure 4). This approach provides several benefits over GSIs, including the selective reduction of Aβ42 without fully blocking γ-secretase activity, thereby minimizing adverse effects on Notch signaling and allowing for an expanded therapeutic range. Subsequent research uncovered that γ-secretase exhibits distinct localization patterns within tissues and performs specific functions in various cell types (Houser et al., 2023; Strope and Wilkins, 2023; Kwak et al., 2022). These findings raise questions regarding whether γ-secretase activity is regulated by other proteins or signaling pathways. Studies have demonstrated that only a fraction of the γ-secretase complex possesses catalytic activity, and the regulation of this activity remains an open question. GSMPs have emerged as key regulators of γ-secretase activity and specificity, enabling rapid responses to cellular signals and environmental changes (Antony et al., 2025). Multiple GSMPs direct the ubiquitous γ-secretase to initiate appropriate signaling under specific conditions. Notably, GSMPs do not directly inhibit γ-secretase activity but instead modulate it, thereby targeting GMSPs to treat AD may mitigate adverse reactions. BSIs, GSIs, GSMs, and GSMPs can all play a certain role in targeting Aβ deposition. The research progress among them is summarized in Table 3.

**TABLE 3:** The comparison of β-secretase 1 inhibitors, γ-secretase inhibitors (GSIs), γ-secretase modulators (GSMs), and γ-secretase modulatory proteins (GSMPs) in Alzheimer’s disease (AD).

Category	Mechanism	Advantage	Side effects	Clinical progression
β-secretase inhibitor	Inhibiting BACE1, blocking APP cleavage at the β-site, and reducing Aβ peptide production.	Significantly reduce Aβ levels in cerebrospinal fluid and theoretically inhibit Aβ production.	Hepatotoxicity and cognitive side effects, affecting other BACE1 substrates	Phase III clinical trials were partially terminated (Chen et al., 2017; Schenk et al., 2012; Bateman et al., 2009)
γ-secretase inhibitor	Directly inhibit γ-secretase activity, block the cleavage of all γ-secretase substrates.	Rapidly reduce total Aβ levels, including Aβ42.	Gastrointestinal toxicity, immunosuppression, and increased skin cancer risk due to Notch pathway inhibition.	Phase III clinical trials were terminated due to toxicity/ineffectiveness (Hur, 2022; Wolfe, 2012)
γ-secretase modulators	Allosteric modulation of γ-secretase selectively reduces Aβ42 and enhances Aβ37/38 levels without affecting total Aβ or other substrates.	Avoid Notch-related toxicity, preserve γ-secretase function.	Early-generation GSMs: poor brain penetration, low efficacy, a narrow therapeutic window, and failed to improve cognition in Phase III trials.	First-generation: failed; Second-generation: some toxicity; New GSMs: preclinical studies (Ji-Yeun, 2022; Raven et al., 2017)
γ-secretase modulatory proteins	Regulating γ-secretase activity or localization, affects Aβ generation.	High targeting specificity; reduces Aβ42 without affecting Notch	Complex mechanism, preclinical test, lack clinical validation.	Preclinical studies, no clinical trials (Kehs et al., 2025; Luo and Li, 2022).

Research on γ-secretase still faces numerous challenges. For example, the mechanism by which GSMs interact with γ-secretase remains unclear. While most studies suggest that GSMs target the PSEN subunit of γ-secretase (Yang et al., 2021), alternative hypotheses propose the formation of a GSM-substrate-γ-secretase ternary complex (Petit et al., 2022). This uncertainty regarding the molecular target hampers the effective optimization of lead compounds. Furthermore, several GSMs have been discontinued from clinical development due to poor BBB penetration and safety concerns such as adverse side effects (Rynearson et al., 2021; Green et al., 2009). These failures underscore the translational gap between animal models and human trials. In addition, the physiological roles of GSM-regulated Aβ37/Aβ38 peptides in humans remain poorly understood, and their potential to cause unintended effects is still undetermined. Furthermore, studies on GSMPs are at an early stage but face multiple challenges. These include determining their precise cellular localization across different cell types, elucidating the mechanisms underlying substrate selectivity, and understanding how specific GSMP subtypes influence substrate processing. Moreover, as protein-based therapeutics, GSMPs are generally large and struggle to cross the BBB, and exogenous proteins may provoke immune responses, compromising both safety and therapeutic efficacy. Finally, biopharmaceuticals typically involve high production costs, complex manufacturing processes, and lower stability compared to small-molecule drugs. Currently, most research efforts are focused on small-molecule GSMs. In contrast, GSMPs represent a promising yet underexplored therapeutic avenue, with unique mechanisms and development hurdles that warrant further investigation. Addressing these gaps could significantly advance our understanding of γ-secretase biology in disease contexts, potentially leading to therapies with improved safety profiles and greater efficacy.
